# Evolution of Thermal Response Properties in a Cold-Activated TRP Channel

**DOI:** 10.1371/journal.pone.0005741

**Published:** 2009-05-29

**Authors:** Benjamin R. Myers, Yaron M. Sigal, David Julius

**Affiliations:** Department of Physiology; University of California San Francisco, San Francisco, California, United States of America; The Rockefeller University, United States of America

## Abstract

Animals sense changes in ambient temperature irrespective of whether core body temperature is internally maintained (homeotherms) or subject to environmental variation (poikilotherms). Here we show that a cold-sensitive ion channel, TRPM8, displays dramatically different thermal activation ranges in frogs versus mammals or birds, consistent with variations in these species' cutaneous and core body temperatures. Thus, somatosensory receptors are not static through evolution, but show functional diversity reflecting the characteristics of an organism's ecological niche.

## Introduction

The ability to sense environmental temperature is fundamental to all metazoans. Transient receptor potential (TRP) cation channels play important roles as detectors of ambient temperature in both vertebrate and invertebrate organisms [1]–[4]. For example, the capsaicin receptor, TRPV1, is expressed by primary afferent somatosensory neurons, where it functions as a detector of noxious heat [5]. A related cation channel, TRPM8, is activated by cold temperatures as well as pharmacological agents that mimic the psychophysical sensation of cold, such as menthol [6], [7]. The thermal activation thresholds for mammalian TRPV1 and TRPM8 are appropriately set to measure ambient temperatures that fall appreciably outside normal core body or skin temperature. Thus, rat, mouse, and human TRPM8 are activated once temperatures drop below 26°C, such that the channel is closed at normal body temperature, but can respond with appropriate intensity to both innocuous and noxious cold stimuli [6], [7]. Indeed, mice deficient in TRPM8 [8]–[10] display pronounced defects in responses to pharmacological “cooling” agents and cold at both cellular and behavioral levels, illustrating that, in mammals, this channel plays a physiologically relevant role in the detection of environmental temperature.

While several key TRP channels that regulate mammalian temperature transduction have been examined in considerable detail, less attention has been devoted to the molecular basis of thermoreception in other animals. Surveys across genome sequences have suggested that TRPM8 channels are found in a wide range of metazoans [11], [12], including those that do not maintain a constant core temperature (poikilotherms) and whose ecological thermal niches differ substantially from those of most homeothermic mammals. Because the process of thermoreception involves the measurement of temperature differences between environment and nociceptor terminal, it is possible that sensory neurons from poikilotherms respond optimally within a temperature range appropriate to their own environment. If so, then alteration of TRPM8 thermal activation properties throughout metazoan evolution could tune cold-sensitive neurons to temperatures most relevant to an animal's ecological niche. However, to date, characterization of TRPM8 channels has been limited to homeothermic species inhabiting relatively similar environments and displaying only modest variations in core body temperature.

Here, we examine native and cloned TRPM8 channels from an aquatic amphibian, the South African clawed frog *Xenopus laevis*. Because this poikilothermic animal lives mostly in ponds and rivers within sub-Saharan Africa, its core body temperature range is substantially colder than that of mammals [13]–[15]. We demonstrate a striking shift in the thermal response properties of *X. laevis* TRPM8 compared to its mammalian and avian counterparts, supporting the notion that the properties of temperature-sensitive TRP channels are under strong evolutionary pressure to conform to a physiologically relevant temperature range.

## Results

**Figure 1:**
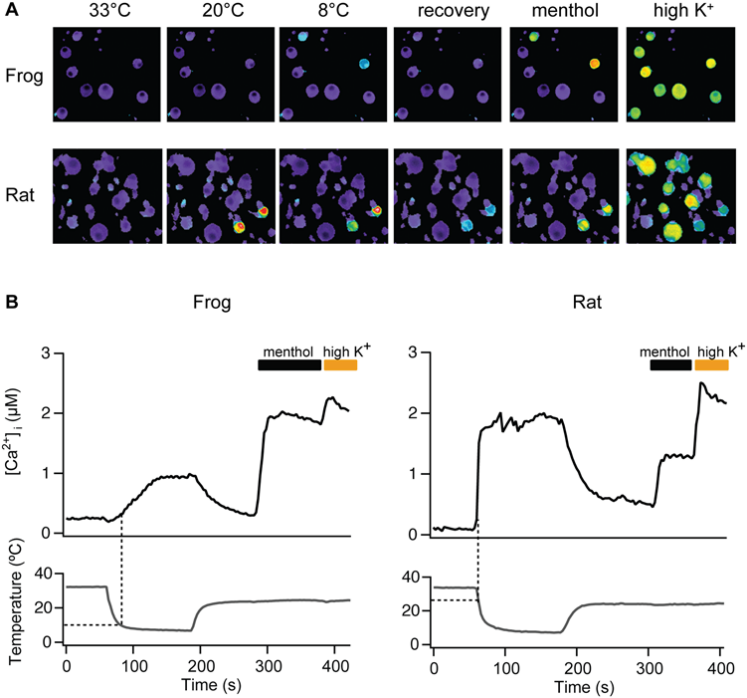
Differences in temperature preference for frog versus rat are reflected in differential cold sensitivity of primary afferent neurons. (A) Dissociated DRG neurons from *X. laevis* frogs or rats were exposed to a cold ramp (33°C–7°C) and responses measured by ratiometric calcium imaging. After a recovery period at 25°C, cells were challenged with menthol (500 µM), followed by high extracellular potassium (70 mM KCl) to depolarize and identify all excitable cells. Note differential sensitivity of frog and rat neurons to 20°C stimulus. (B) Averaged trace of calcium signals from menthol-resopnsive rat or frog DRG neurons stimulated as described in (A). Dotted lines indicate respective cold activation thresholds (9.6±0.6°C for frog and 25.4±1.3°C for rat) (n = 30–40 cells). Cells showing an increase in intracellular calcium greater than five standard deviations above baseline fluctuations were taken as positive responders.

To learn more about the molecular basis for detection of cold temperatures in poikilothermic animals, we measured temperature responsiveness of sensory neurons dissociated from dorsal root ganglia (DRG) of *X. laevis*, which tolerates a range of temperatures (14–32°C) and displays a preferred average of 22.4°C, substantially colder than that for rodents (28°C as measured by thermal gradient preference) [13], [16]. First, we found that 23.7% of frog neurons were excited by menthol, the vast majority of which also responded to decreases in ambient temperature (Fig. 1A), resembling the distribution seen with rat DRG neurons. Interestingly, frog neurons responded to cold stimuli with a thermal activation threshold of 9.6±0.6°C, compared with a much warmer threshold of 25.4±1.3°C for rat (n = 594 and 1548 neurons, respectively) (Fig. 1B), suggesting that differential thermal preferences of these species are, in fact, manifest at the level of the primary afferent sensory neuron.

**Figure 2:**
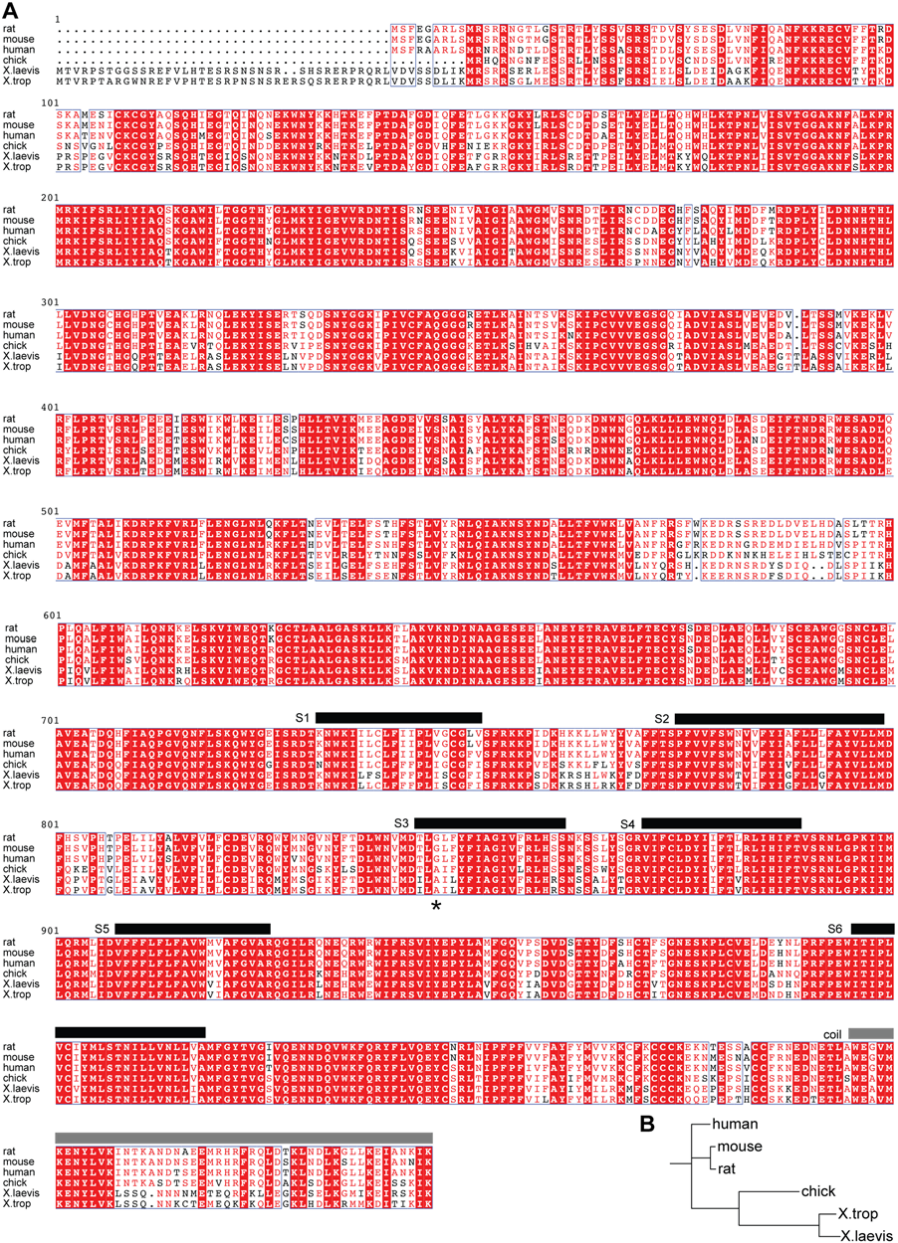
Sequence comparison of TRPM8 species orthologs. (A) Previously described rat, human, and chicken TRPM8 sequences were aligned with the full-length sequences of *X. tropicalis* and *X. laevis* TRPM8 (this study) using MultAlin and ESPript. The locations of predicted transmembrane helices [6] and the C-terminal coiled-coiled assembly domain [25] are shown as black and gray bars, respectively. The asterisk indicates the polymorphic residue previously shown to determine TRPM8 icilin sensitivity [17]. (B) Phylogenetic tree indicating the evolutionary relationship between TRPM8 ortholog sequences.

To determine whether the observed difference in sensory neuron cold sensitivity is attributable to alterations in the intrinsic thermal responsiveness of TRPM8 channels, we isolated cDNAs encoding TRPM8 from *X. laevis* sensory ganglia and characterized their functional properties when expressed heterologously. As previously reported [11], the genome of frogs contains two distinct open reading frames that are homologous to mammalian TRPM8, displaying 74 or 65% amino acid identity to the rat sequence (xlTRPM8 and xlTRPM8b, respectively) (Fig. 2). Only xlTRPM8 produced functional cold- and menthol-gated channels upon heterologous expression (see below). Moreover, co-expression of xlTRPM8 with xlTRPM8b did not substantially alter responses observed with xlTRPM8 alone (not shown). For the purposes of this study, we therefore focused our attention on xlTRPM8.

**Figure 3:**
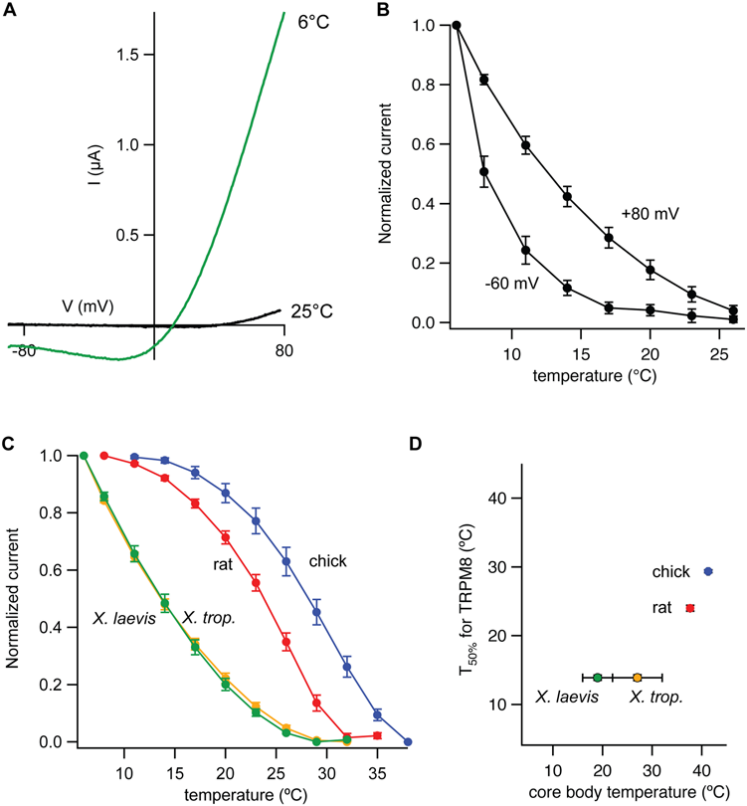
Cloned *Xenopus* TRPM8 channels show alterations in thermosensitivity compared to their mammalian and avian counterparts. (A) Current-voltage relation from a two-electrode voltage clamp recording of xlTRPM8-expressing oocytes under basal or cold-stimulated conditions. Note the strong outward rectification of the cold-evoked current. (B) Normalized inward (−60 mV) and outward (+80 mV) cold-evoked currents for xlTRPM8. The current obtained at a given temperature was normalized to the maximum cold-evoked current (obtained at 6°C) obtained at each potential. (C) Normalized current-temperature plots (at +80 mV) for chicken (blue), rat (red), *X. laevis* (green) and *X. tropicalis* (orange) TRPM8, yielding half-maximal activation temperatures of 29.35±0.21°C (chicken), 24.00±0.43°C (rat), 13.89±0.39°C (*X. laevis*), and 13.90±0.44°C (*X. trop.*). (D) Plot of species core body temperature (obtained from previously published measurements) versus experimentally determined temperature of half-maximal TRPM8 cold activation. Core temperatures were based on previously published values (for *Xenopus*, core temperatures were estimated as the arithmetic mean and error bars indicate the full range of tolerated temperatures) [14], [18], [19].

xlTRPM8 was robustly activated by cold, and resultant currents displayed characteristic outward rectification and voltage dependence (Fig. 3A,B). However, unlike its mammalian and avian counterparts [6], [7], [17], the frog channel showed little or no basal activity at room temperature (25°C) and required substantially lower temperatures for activation at physiological membrane potentials (Fig. 3B). Indeed, comparison of TRPM8 orthologs revealed a dramatic leftward shift in temperature response curves for xlTRPM8 versus rat or avian receptors (10°C and 15°C shifts, respectively, in half-maximal activation temperatures) (Fig. 3C). To determine whether this divergence in cold sensitivity is unique to xlTRPM8, we cloned and characterized TRPM8 from another frog species, *Xenopus tropicalis*. When expressed heterologously, *X. tropicalis* TRPM8 exhibited a thermal response profile indistinguishable from that of the *X. laevis* channel (Fig. 3C). While the preferred temperature range for *X. laevis* is reported to be somewhat lower than that for *X. tropicalis* [14], both frogs exist in comparable niches where average environmental (and thus body) temperature is substantially lower than core body temperature of mammals or birds. As previously described [17], we also observed a slightly higher thermal activation range for chicken TRPM8 compared with the rat channel (Fig. 3D), consistent with the elevated cutaneous and core body temperature of birds compared to mammals [18], [19]. Taken together, these data point to a correlation between species body or skin temperature and the thermal response range of the cognate TRPM8 channel (Fig. 2D).

**Figure 4:**
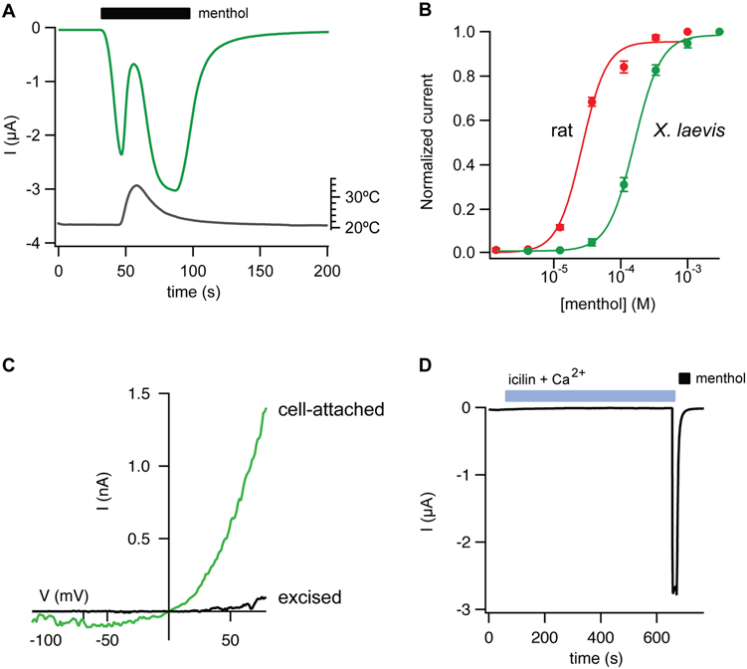
*X. laevis* TRPM8 is activated by menthol but not icilin. (A) In oocytes expressing xlTRPM8, application of menthol (1 mM) evoked inward currents (green trace) that were suppressed by a rise in bath temperature (gray trace). Holding potential was −60 mV. (B) Cell-attached patches from oocytes expressing xlTRPM8 (n = 4) but not uninjected oocytes (n = 3) displayed a strongly rectifying current (green trace) when the pipette solution contained 500 µM menthol. Formation of the inside-out configuration resulted in rapid current rundown (black trace, 90 seconds after patch excision.) (C) Concentration-response relation for menthol-evoked currents (at +80 mV) from rat (red) or *X. laevis* (green) TRPM8-expressing oocytes. (D) Application of 10 µM icilin in the presence of 2 mM extracellular Ca^2+^ failed to activate xlTRPM8, while 1 mM menthol evoked robust inward currents.

We also evaluated the responsiveness of *X. laevis* TRPM8 to pharmacological agonists. Thus, menthol produced robust activation of xlTRPM8 that was suppressed by a moderate rise in bath temperature (Fig. 4A), as observed for mammalian and avian receptors, where ligand and temperature gating show allosteric coupling. This observation demonstrates that the frog channel, like its counterparts from homeothermic species, can be modulated over a wide dynamic temperature range irrespective of its normal physiological thermal threshold. Thus, the frog channel showed decreased menthol sensitivity compared to rat TRPM8 (Fig. 4B; EC50 = 160.1±5.8 µM and 26.4±3.6 µM, respectively), in keeping with its lower basal activation at room temperature. Consistent with this trend, chicken TRPM8 displays higher menthol sensitivity compared to the mammalian or frog orthologs [17]. In cell-attached membrane patches from oocytes expressing xlTRPM8, menthol-evoked currents showed strong outward rectification and ran down rapidly upon excision to the inside-out configuration (Fig. 4C), as previously reported for rat TRPM8 [20]. Whereas mammalian and avian TRPM8 are robustly activated by menthol, only the former is sensitive to the synthetic “super-cooling” agent icilin. The structural determinant of this pharmacological difference is a polymorphism within the putative cytoplasmic loop connecting the second and third transmembrane domains [17]. In this regard, xlTRPM8 resembles the chicken channel in that it is icilin insensitive (Fig. 4D) and aligns to the chicken sequence at this critical residue (A841 and A796 in xlTRPM8 and cTRPM8, respectively; Fig. 2A). Thus, consistent with previous observations [17], activation by icilin appears to be unique to mammalian TRPM8. We conclude that xlTRPM8 is a bona fide menthol and cold receptor, but with properties that distinguish it from mammalian and avian orthologs.

## Discussion

In this study, we report the cloning and characterization of temperature-sensitive TRP channel orthologs from two cold-blooded aquatic frogs whose optimal environmental temperatures are substantially lower than those of mammals [13]–[15]. In comparing the thermal activation properties of several TRPM8 orthologs, we found a dramatic shift in the range of temperatures at which *Xenopus* TRPM8 channels are gated, such that significant activation occurs only at temperatures much colder than for rat or chicken TRPM8. This shift in thermal activation corresponds well with the reduction in core body temperature (and, by extension, the skin temperature at the nociceptor terminal) that would be expected in this poikilothermic species. Indeed, for the TRPM8 species orthologs examined in this study, we found a striking correlation between the species' core body temperature and the temperature required to observe half-maximal TRPM8 activation, suggesting that the biophysical properties of TRPM8 in each species are tuned to the most appropriate temperature range.

We also found that *X. laevis* TRPM8 responds strongly to the plant-derived cooling agent menthol and (like its avian counterpart) is insensitive to icilin, raising the possibility that icilin mimics an endogenous TRPM8 modulator that plays a physiological role in mammals, but not amphibians or birds. This would resemble the differential sensitivity of mammalian and avian TRPV1 channels to both exogenous and endogenous ligands (capsaicin and anandamide, respectively) that likely activate the channel by binding to a common site [21].

Within visual and chemosensory systems, stimulus detectors (receptors) undergo great functional diversification as organisms evolve to inhabit a wide range of ecological niches [22], [23]. Our findings demonstrate that genes encoding somatosensory receptors display the same capacity for adaptation to species' environmental conditions. Specifically, we have shown that a cold receptor can be tuned to respond within a temperature range most relevant to the normal resting temperature of the primary afferent nerve terminal, whether determined by an internally regulated core body temperature or the environmental milieu. Although the frog TRPM8 channel is clearly distinct from its mammalian or avian counterparts by virtue of an extremely low thermal activation threshold, it remains to be determined whether this represents a prototypical TRPM8 channel for all poikilothermic species, or whether the full complement of poikilothermic TRPM8 genes exhibits a continuum of thermal response properties that varies strictly with organismal body temperature. It may also be interesting to examine species that experience substantial variations (short- or long-term) in environmental temperature, as there may be corresponding changes in TRPM8 expression and/or function that allow for optimal temperature detection under such circumstances. In any case, our data show that the functional properties of thermoreceptors are not static through evolution, but rather reflect a natural diversity in thermoregulatory processes.

## Materials and Methods

### Culture and live-cell imaging of *X. laevis* sensory neurons

Frog neurons were prepared as previously described [24] with minor modifications. Briefly, dorsal root ganglia were dissected from juvenile 2.5–5 cm *X. laevis* (Nasco, Inc.) into frog neuron medium (50% Leibovitz L-15, 49% normal Frog Ringer's (in mM: 115 NaCl, 2.6 KCl, 10 HEPES, 5.5 glucose, 2 CaCl_2_, pH 7.6), and 1% serum). The connective tissue was removed and each ganglion minced into several pieces, transferred to Ca^2+^/Mg^2+^ free Hank's Balanced Salt Solution (HBSS) containing 1 mg/ml Collagenase P (Roche, Inc.), and digested for 45 minutes at room temperature. Neurons were pelleted at 500×*g* and further digested for 30 minutes in 0.25% (w/v) saline-based trypsin solution at room temperature. Neurons were triturated to a uniform suspension using a fire-polished glass pipette, resuspended in frog neuron medium, and layered onto 20% Percoll in frog Ringer's solution. Neurons were pelleted at 1000×*g*, washed in frog neuron medium, and plated onto poly-D-lysine-coated coverslips (BD Biosciences). Neurons were cultured in frog neuron medium overnight on the bench, loaded for one hour with 10 µM fura2-AM+0.01% pluronic acid in normal frog Ringer's solution, and subject to ratiometric calcium imaging essentially as previously described [8] except that frog Ringer's solution was used in place of standard mammalian Ringer's solution. Dissection, culture, and calcium imaging of rat dorsal root ganglia were as previously described [6], [8]. Acute collection of *X. laevis* and rat tissues were performed according to our laboratory protocol (#AN080281-01C) using animals obtained from our frog and rodent colonies, and all procedures for animal husbandry and euthanasia were approved by the UCSF Institutional Animal Care and Use Committee.

### Molecular biology

Partial sequences for *X. tropicalis* TRPM8 and TRPM8-b were obtained by searching ENSEMBL and JGI databases, and from a previous bioinformatic study [11]. Total RNA was extracted from *X. laevis* trigeminal and dorsal root ganglia or whole *X. tropicalis* stage 43 tadpoles using TRIZOL (Invitrogen) and reverse transcribed using SuperScript II MMLV-RT (Invitrogen). *X. tropicalis* tissue was dissected by Jessica Lyons and Richard Harland (University of California, Berkeley) using animals from their laboratory frog colony; tissue was provided to us following dissection. Internal PCR products for *X. laevis* TRPM8 and TRPM8-b were obtained using PCR primers designed to amplify conserved regions within each gene (based on rat, human, chick, and *X. tropicalis* genome sequences), and the resulting sequence information was used to obtain complete transcript sequences using 5′ and 3′ SmartRACE (Clontech.) Full-length genes were captured by TA-cloning.

Primer pairs to amplify full-length genes were as follows:

*X. laevis* TRPM8, 5′-CAGTGTGCTCTGGTCTTAGCTTTAC-3′

5′-TCACTTTATCTTGCTTCTGATTTCC-3′;

*X. tropicalis* TRPM8

5′ATGACTGTCAGACCCACGGCGAGAGGATGGAACAGAGAGTTTGTGCCTCACACAGAATCA-3′ and

5′-GGTCCAGGTAAGGTCAATGGTCTTC-3′;

*X. laevis* TRPM8-b, 5′-CTGATAGACATCTTGGATACTTCAG-3′ and 5′-CATCTATCCCGCTCCAGGAATAGTGG-3′;

*X. tropicalis* TRPM8-b, 5′-TCAGGAATGAATTACCAACATGACGTGG-3′ and 5′-CATATCCCTCTCCAGGAATAGCGG-3′.

Genes were subcloned into the combined mammalian/oocyte expression vector pMO. For in vitro RNA transcription, channel constructs were linearized, transcribed (mMessage Machine T7 transcription kit, Ambion), and resuspended at a final concentration of 1 µg/µl. GenBank accession numbers are as follows: FJ948757 (*X. laevis* TRPM8), FJ948758 (*X. tropicalis* TRPM8), FJ948759 (*X. laevis* TRPM8b), FJ948760 (*X. tropicalis* TRPM8b).

### Frog oocyte culture and electrophysiology

Surgically extracted oocytes from *X. laevis* (Nasco) were cultured and analyzed 2–7 days post-injection by two-electrode voltage clamp (TEVC) as previously described [17]. For TEVC analysis, bath solution contained (in mM): 120 CsCl, 10 HEPES, 2 MgCl_2_-6H_2_O, 1 EGTA, pH 7.4 For patch clamp analysis, both the bath and pipette solutions contained (in mM): 140 CsCl, 10 HEPES, 1 MgCl_2_-6H_2_O, 1 EGTA, pH 7.4. Macropatches were excised from devitellinized oocytes using pipettes with resistances of 0.6–1.0 MΩ and currents were recorded with an Axopatch 200B amplifier (Molecular Devices) using a 180 ms voltage ramp from −120 mV to +80 mV delivered once per second. Currents were recorded at 5 kHz, filtered at 2 kHz, and analyzed with pClamp 10 (Molecular Devices). Temperature ramps were generated with a custom-made Peltier device (Reid-Dan Electronics) that cooled the flowing perfusate stream. Temperature was measured using a thermistor placed adjacent to the cell. Menthol and icilin stocks were dissolved in DMSO and diluted into recording solution immediately prior to experiments.
